# Evaluation of dried blood spot samples for screening of hepatitis C and human immunodeficiency virus in a real-world setting

**DOI:** 10.1038/s41598-018-20312-5

**Published:** 2018-01-30

**Authors:** Sonia Vázquez-Morón, Pablo Ryan, Beatriz Ardizone-Jiménez, Dolores Martín, Jesus Troya, Guillermo Cuevas, Jorge Valencia, María A. Jimenez-Sousa, Ana Avellón, Salvador Resino

**Affiliations:** 1 Laboratorio de Referencia e Investigación en Hepatitis Víricas, Centro Nacional de Microbiología, Instituto de Salud Carlos III, Majadahonda, Madrid, Spain; 2 Hospital Universitario Infanta Leonor (HUIL), Vallecas, Madrid, Spain; 3 BR Salud, Madrid, Spain; 4 Madrid Positivo, Madrid, Spain

## Abstract

Both hepatitis C virus (HCV) infection and human immunodeficiency virus (HIV) infection are underdiagnosed, particularly in low-income countries and in difficult-to-access populations. Our aim was to develop and evaluate a methodology for the detection of HCV and HIV infection based on capillary dry blood spot (DBS) samples taken under real-world conditions. We carried out a cross-sectional study of 139 individuals (31 healthy controls, 68 HCV-monoinfected patients, and 40 HCV/HIV-coinfected patients). ELISA was used for anti-HCV and anti-HIV antibody detection; and SYBR Green RT-PCR was used for HCV-RNA detection. The HIV serological analysis revealed 100% sensitivity, specificity, positive predictive value (PPV), and negative predictive value (NPV). The HCV serological analysis revealed a sensitivity of 92.6%, specificity of 100%, PPV of 100%, and NPV of 79.5%. Finally, the HCV-RNA detection test revealed a detection limit of 5 copies/µl with an efficiency of 100% and sensitivity of 99.1%, specificity of 100%, PPV of 100%, and NPV of 96.9%. In conclusion, our methodology was able to detect both HCV infection and HIV infection from the same DBS sample with good diagnostic performance. Screening for HCV and HIV using DBS might be a key strategy in the implementation of national programs for the control of both infections.

## Introduction

Hepatitis C virus (HCV) infection is a major health problem worldwide. Approximately 71 million persons live with HCV infection, and around 80% are undiagnosed^1^. Infection by human immunodeficiency virus (HIV) continues to be a major public health issue, with a global prevalence of 0.8% among adults, of whom around 30% are undiagnosed^2^. Both HIV and HCV share routes of transmission, and their diagnosis is a priority in public health strategies throughout the world^1,2^. On the one hand, HIV infection is diagnosed increasingly frequently in resource-limited settings through implementation of HIV testing programs; however, HIV testing is still not reaching some communities (eg, people who inject drugs [PWID], sex workers, homeless persons), resulting in late presentation^3^. On the other hand, diagnosis of HCV remains problematic for many low-income countries and difficult-to-access populations in developed countries^4^, where most individuals remain unaware of their HCV status until advanced stages of the disease^5–7^.

Traditionally, diagnosis of HIV and HCV infection has been based on the detection of antibodies, antigens, and the viral genome in blood samples obtained by routine venous blood collection^5,8^. However, the use of capillary whole blood in dried blood spot (DBS) specimens may be an excellent alternative in both serological and nucleic acid testing (NAT) assays for the screening of HIV and HCV infection, since these approaches are less invasive, samples can be stored and transported without refrigeration, and highly trained personnel are not necessary^5,9,10^. Therefore, DBS may facilitate diagnosis of HIV and HCV infection in settings where access to screening is difficult. In fact, the World Health Organization (WHO) indicates that future directions and innovations in HIV and HCV testing should include validation of DBS specimens with various commercial serological and NAT assays (multiplex and polyvalent platforms) for integrated testing of HCV and HIV^5^.

In this study, our aim was to develop and evaluate a methodology that makes it possible to detect HCV and HIV infection from capillary DBS samples under real-world conditions.

## Results

### Characteristics of the study population

The main characteristics of the individuals included in the study are shown in Table 1. We studied 139 subjects who were stratified into groups: non-infected volunteers and HCV-infected patients (HCV-monoinfected and HCV/HIV-coinfected). HCV-infected patients were infected by genotypes 1 to 5. HCV/HIV-coinfected patients were infected by HIV-1.

**Table 1:** Main characteristics of the population studied, according to HCV infection status and HIV coinfection status.

	Non-infected volunteers	HCV-monoinfected patients	HIV/HCV-coinfected patients
No.	31	68	40
Male	12 (38.7%)	45 (66.2%)	36 (90%)
Age	35.0 (26.0; 51.5)	51.8 (45.4; 58.5)	46.2 (42.1; 50.6)
HCV genotype			
1a	—	17 (25%)	19 (47.5%)
1b	—	25 (36.8%)	3 (7.5%)
2	—	6 (8.8%)	1 (2.5%)
3	—	10 (14.7%)	11 (27.5%)
4	—	9 (13.2%)	6 (15%)
5	—	1 (1.5%)	0 (0.0%)
Log_10_ HCV viral load (IU/ml)	—	6.00 (5.31; 6.38)	6.50 (5.48; 6.84)
CD4+ T-cells/mm^3^	—	—	536 (373; 682)
HIV viral load <37 copias/ml	—	—	29 (72.5%)

Values are shown as absolute count (percentage) or median (percentile 25; percentile 75).

Abbreviations: HCV, hepatitis C virus; HIV, human immunodeficiency virus; p25, 25^th^ percentile; p75, 75^th^ percentile.

### HIV and HCV serological analysis

The measures of diagnostic accuracy are summarized in Table 2. All 40 DBS samples from 40 HIV-infected patients were positive for HIV antibodies; therefore, the sensitivity was 100%. Moreover, 99 DBS samples from 99 HIV-negative subjects were negative for HIV antibody; therefore, the specificity was 100%. Additionally, PPV was 100%, and NPV 100%. We could not obtain the PLR or NLR value, because there were no false positives or negatives, respectively.

**Table 2:** Summary of diagnostic accuracy of assays for detection of anti-HIV antibodies, anti-HCV antibodies, and HCV-RNA using DBS samples.

Screening test	TP	FP	TN	FN	Se (95%CI)	Sp (95%CI)	PPV (95%CI)	NPV (95%CI)	LR + (95%CI)	LR– (95%CI)
Anti-HIV antibodies	40	0	99	0	100% (91.2%; 100%)	100% (96.3%; 100%)	100% (91.2%; 100%)	100% (96.3%; 100%)	N/A	N/A
Anti-HCV antibodies	100	0	31	8	92.6% (86.1%; 96.2%)	100% (89%; 100%)	100% (96.3%; 100%)	79.5% (64.5%; 89.2%)	N/A	0.07 (0.04; 0.14)
HCV-RNA	107	0	31	1	99.1% (94.9%; 99.8%)	100% (89%; 100%)	100% (96.5%; 100%)	96.9% (84.3%; 99.4%)	N/A	0.01 (0.00; 0.07)

Abbreviations: FN, false negative; FP, false positive; TN, true negative; TP, true positive; LR+, positive likelihood ratio; N/A, not available LR−, negative likelihood ratio; NPV, negative predictive value; PPV, positive predictive value; Se, sensitivity; Sp, specificity.

Of the 108 HCV-infected patients, 100 DBS samples were positive and 8 DBS samples were false negatives; therefore, the sensitivity was 92.6%. The 8 false negatives were retested using the INNO-LIA™ HCV Score test, which revealed 4 positive samples, 3 negative samples, and 1 indeterminate result. Moreover, 31 DBS samples from the 31 HCV-negative subjects were negative for HCV antibody (100% specificity). Additionally, PPV was 100%, NPV was 79.5%, and NLR was 0.07. We could not obtain the PLR value, because there were no false positives.

### HCV-RNA detection by SYBR Green RT-PCR in DBS samples

The assay showed a detection limit of 5 copies/µl with an efficiency of 100%. The amplification products were confirmed by visualizing a 224-bp band for HCV and a 277-bp band for IC-RNA. The melting curves obtained showed a Tm of 85.73 ± 0.54 °C for the HCV amplicon and a Tm of 88.3 ± 0.5 °C for the IC-RNA.

The HCV-RNA assay revealed that of the 108 patients who tested positive for HCV-RNA in plasma samples, HCV was detected in 107 DBS samples by SYBR Green RT-PCR One Step, with 1 false negative (Table 2); therefore, the sensitivity was 99.1%. Moreover, 31 DBS samples from 31 HCV-negative subjects were negative for HCV-RNA; therefore, the specificity was 100%. Additionally, PPV was 100%, NPV was 96.9%, and NLR was 0.01. We could not obtain the PLR value, because there were no false positives.

## Discussion

DBS samples are useful for screening for HCV and HIV infection, especially in resource-limited areas or difficult-to-reach populations. There are a large number of articles on this topic, but there are few data that explore the diagnostic accuracy of the techniques used for HCV screening under real-world conditions. In this study, we developed and evaluated a method for the detection of HCV infection (HCV-RNA and anti-HCV antibodies) and HIV infection (HIV antigen/antibodies) from the same capillary DBS sample. We achieved good diagnostic performance for both HCV and HIV infection, finding high sensitivity, specificity, NPV, and PPV values.

Current guidelines for the diagnosis of HCV infection include an initial test for anti-HCV antibody detection (indirect tests) as a first step, followed by a NAT test for the detection of HCV-RNA (direct tests) in serum or plasma, if the initial HCV antibody test is reactive^7,10^. However, it is important to note that, in the future, detection of active HCV infection (HCV-RNA or core antigen) will be essential for HCV screening, since most of the HCV-infected individuals will be treated. Additionally, populations from low-income countries or difficult-to-access populations (eg, drug users, homeless persons) may have a high prevalence of HCV infection, and around 15–30% clear HCV infection spontaneously within 6 to 12 months of the initial exposure^11^. Thus, the percentage of patients with positive HCV antibody detection and negative HCV-RNA will be greater, and communication of active HCV infection will eliminate the need to return for confirmatory testing in the case of seropositive individuals^11^. Consequently, anti-HCV antibody detection will not be the most appropriate tool for HCV screening, especially in difficult-to-access populations.

In our study, testing for anti-HCV antibodies was performed in capillary DBS samples. We detected 8 false negatives; therefore, sensitivity (92.6%) and NPV (79.5%) were lower than estimated in several previously published studies of DBS samples^12–17^, but similar or higher than in other studies^18–22^, probably owing to the lower amount antibody titers in DBS samples than in serum samples. Consequently, an eluted volume of DBS higher than that recommended for serum samples could be necessary to increase their efficiency, as indicated elsewhere^12,21,23^. In our study, the eluted volume of DBS was not increased, since we wished to calculate the baseline diagnostic accuracy of the techniques used and to verify whether they were suitable for application under real-world conditions. In addition, these discrepant results between studies could also have been due to the different technologies used. In fact, in our study, the confirmatory antibody test (INNO-LIA™ HCV Score) made it possible to detect anti-HCV antibodies from the same eluted DBS samples more easily than with the Murex anti-HCV version 4.0 kit.

As for the methodology used in HCV-RNA detection, we found that diagnostic performance was better for HCV-RNA detection than anti-HCV antibody detection, although our assay for HCV-RNA detection did not show relevant differences in diagnostic performance with respect to previous studies^17,24–27^. Of note, we included patients infected with different HCV genotypes, whereas other studies analyzed a limited number of HCV genotypes or, in some cases, information on genotypes was missing^22,25–28^. This detail is very important, given the high variability of HCV, which could affect detection of the virus. Moreover, HCV levels are around 1.50–2.5 log IU/ml higher in serum samples than in DBS samples^16,17^, with DBS samples presenting a lower sensitivity for HCV-RNA detection, although this difference may vary according to the DBS elution procedure, RNA extraction method, and HCV detection technique used^4,17^. Nevertheless, in our study, the diagnostic performance obtained with SYBR Green RT-PCR was scarcely affected by the HCV genotype, as indicated by the high sensitivity and specificity values obtained, probably owing to the use of degenerated primers to cover all possible HCV genotypes.

As discussed above, the sensitivity of DBS for HCV-RNA detection at the lower end of the dynamic range is worse than that of plasma or serum^16,17^; consequently, DBS is not the most appropriate method for monitoring the current HCV treatment^4,17^. However, this would not invalidate its use in untreated HCV-infected patients, who usually have a higher viral load than treated patients^4^. In addition, patients who experience relapses during or after treatment usually have high HCV viral loads that are above the limit of detection of the tests applied to DBS samples (150-250 IU/ml)^29^. Therefore, given that the main purpose of DBS samples will be for HCV screening without the need to determine viral load, the methodology validated here is appropriate.

Current HIV screening guidelines recommend a combined assay for the detection of antigen/antibody^8^. When the results are positive, a supplemental HIV-1/2 antibody differentiation assay must be performed, and the HIV-RNA test must be used to resolve negative or indeterminate supplemental results. We found good diagnostic performance in the serological HIV screening assay in the same capillary DBS sample, since the sensitivity and specificity were very high (100%), thus preventing losses in HIV detection actions. Our results were similar to previously published findings^26,30–32^, leading us to consider that performing the HIV assay with the same DBS sample is good for HIV screening, as the main objective should be to have no false negatives^26,30–32^. Therefore, our study demonstrates a high diagnostic performance for HIV screening in DBS samples.

We did not assess HIV RNA levels, HCV viral genotype, and HCV core antigen level in DBS samples, since both serological and NAT assays are enough for the screening of HIV and HCV infection^33^. Moreover, in the coming future with the use of newer and pan-genotypic DAAS, the use of these virological parameters will less important. Moreover, as we have commented previously, our aim was to develop and evaluate a methodology for HCV screening under real-world conditions. Thus, we only evaluated the cut-off point established by the manufacturer of ELISA kit because it is going to be the easiest to implement and the one with fewer problems of interpretation when it is used in laboratories under real-world conditions.

Finally, note that the methodology developed for HCV RNA detection was validated by comparison with the results obtained in the hospital’s laboratory with standard reference tests (our gold standard), in order to know the accuracy of the homemade Sybr RT-PCR using DBS samples under real-world conditions. Furthermore, it must be taken into account that the need for HCV screening involves actions in developed countries and in low-income countries, in which advanced analysis equipment may not be available. Additionally, the cost reduction is important in order to achieve the objective stablished by WHO. Therefore, our purpose was to have a more affordable technique since its greater applicability would be to screen the active HCV infection in any situation.

In conclusion, our methodology detected both HCV and HIV infection in the same DBS sample with good diagnostic performance. Both HCV and HIV screening using DBS could prove useful in the implementation of national programs for the control of both infections in less developed countries and in high-risk populations.

## Material and Methods

### Study design

We carried out a cross-sectional study of 139 individuals from Infanta Leonor University Hospital (HUIL) (Madrid, Spain) between January 2016 and April 2017. The study was conducted in accordance with the Declaration of Helsinki, and patients gave their written informed consent to participate. The Institutional Review Board and the *Instituto de Salud Carlos III* (ISCIII) Research Ethics Committee approved the study.

Subjects were included in 3 mutually exclusive groups: 31 non-infected volunteers (HIV–/HCV–) as the control group, 40 HCV/HIV-coinfected patients, and 68 HCV-monoinfected patients. Clinical and epidemiological data were obtained from medical records.

### Data collection

Study data were collected and managed using Research Electronic Data Capture (REDCap), which is hosted at “Asociación Ideas for Health”^34^.

### DBS samples

DBS samples were collected by finger prick using Whatman 903^®^ cards, and venous blood samples were collected in parallel to obtain plasma samples. DBS samples were sent to the National Microbiology Centre for processing, and plasma/serum samples were tested at HUIL. Spots from DBS cards were dried at room temperature (around 25 °C) for 4 hours and kept in individual zipped plastic bags with a drierite desiccant. Next, DBS samples were stored at 4 °C before being sent to the National Microbiology Center on a monthly basis for detection of HCV and HIV.

### DBS-based anti-HCV and anti-HIV elution and serological assays

Two 6-mm discs were punched from the DBS cards and added to 1200 µl of solution with 0.05% Tween in PBS at 25 °C and centrifuged at 1200 rpm for 30 minutes. Samples were then incubated overnight at 4 °C. The next day, the DBS eluate was stored at −80 °C until analysis. DBS eluates were tested for anti-HCV antibodies and the anti-HIV antigen/antibody combination using the Murex anti-HCV kit, version 4.0 (DiaSorin, Saluggia, Italy) and Murex HIV Ag/Ab Combination Kit (DiaSorin, Saluggia, Italy), respectively, following the manufacturer’s instructions, on an ETI-Max 3000 instrument (DiaSorin, Saluggia, Italy). We used 20 µl for the HCV assay and 100 µl for the HIV assay with pretreated samples. False negatives obtained from DBS samples were confirmed using the INNO-LIA™ HCV Score (Innogenetics, Ghent, Belgium).

### DBS viral RNA extraction

Two 6-mm discs were punched from the DBS cards and pretreated in 1100 µl of ATL buffer (Qiagen, Hilden, Germany) at 56 °C, and centrifuged for 15 minutes at 1200 rpm. Three different protocols were tested using 200 µl, 300 µl, and 400 µl of the pretreated sample in order to know the best conditions for viral RNA extraction. Finally, viral HCV-RNA was extracted from the 300-µl pretreated samples using a commercial mini kit DSP Virus/Pathogen (Qiagen, Hilden, Germany) on a customized protocol in the QIAsymphony instrument. A 3-µl volume of internal control (IC-RNA) with 80,000 copies/µl (Qiagen, Hilden, Germany) was added to each sample in order to control extraction and further amplification. The final elution volume for viral RNA was 60 µl, which was stored until use at −80 °C.

### SYBR Green RT-PCR for HCV detection

Single-step retro-transcription and primary amplification were performed using the Quantitec SYBR Green RT-PCR One Step kit (Qiagen, Hilden, Germany). Several primer pairs were tested to characterize the melting curve for IC-RNA and HCV under different annealing temperatures and concentrations of primers, dNTP and salt. Finally, 8 µl of viral RNA extract was added to the mixture, which contained the following: 12.5 µl of 2X SYBR Green PCR Master Mix; 0.4 µl of dNTPs mix 10 mM; 0.2 µl of MgCl_2_ 25 mM; 0.5 µl of forward primer HCVS_F 5′GYCTAGCCATGGCGTTAGTAYGAG3′ and reverse primer HCVS_R 5′CCCTATCAGGCAGTACCRCAAG3′ and 0.3 µl of IC-11F 5′CAGCCACAACGTCTATATCATG3′ and IC-10R 5′CTTGTACAGCTCGTCCATGC3′ (internal control primers), each at a concentration of 40 µM; 0.25 µl of RT-Enzyme mix; and nuclease-free water to a final volume of 17 μl. All reagents except primers (Sigma), MgCl_2_ (Roche), and dNTPs (Qiagen, Hilden, Germany) were supplied with the kit. Amplification was performed in a RotorGene® device (Qiagen, Hilden, Germany), which was programmed for a first retro-transcription step of 30 min at 50 °C, followed by 15 min at 95 °C for reverse transcriptase inhibition and cDNA denaturation, 40 repetitive cycles of 30 sec at 94 °C, 30 sec at 60 °C, 30 sec at 72 °C, and 15 sec at 81 °C with single fluorescence detection in this step in order to avoid primer-dimer artifacts. After the amplification cycles, a melting curve ranging from 81 °C to 95 °C was generated. PCR products were visualized on a QIAxcell instrument (Qiagen, Hilden, Germany) together with a size marker (50–800 bp) using the OM500 protocol for 20 sec to allow for sample injection time. Positive samples showed a specific band size of 224 bp for HCV and 277 bp for IC-RNA. Standard precautions were taken to avoid carryover contamination. Pipetting was performed with aerosol-resistant tips, and different biosafety cabinets were used for extraction, mixing, and SYBR RT-PCR. Amplicons were detected in a different room. The efficiency of the technique was evaluated using 10 serial dilutions from a DBS sample obtained from a genotype 3 HCV-infected patient with a viral load of 45,975 copies/µl.

### Laboratory test for diagnosis of HCV and HIV infection (gold standard)

HCV infection and HIV infection were diagnosed using the standard assays used at HUIL, which were considered the gold standard.

HCV infection was confirmed in all patients using the enzyme-linked immunosorbent and PCR assays. Serum samples were tested for HCV antibody using the ADVIA Centaur® HCV assay. Plasma samples were tested for HCV-RNA detection using the VERSANT kPCR Molecular System platform (Siemens) and the VERSANT HCV RNA 1.0 Assay kit (kPCR) following the manufacturer’s instructions. Results were reported as international units per milliliter (IU/ml), with a lower limit of detection of 13 IU/ml. HCV genotype was determined by amplification and reverse transcription using the HCV Amplification 2.0 Assay Line Probe Assay (LiPA) Kit in a SimpliAmp TM Thermal Cycler followed by reverse hybridization and detection by the HCV Genotype 2.0 Assay Line Probe Assay (LiPA) in an Auto-LiPA 48 Genotyping Instrument.

Serum samples were also tested for HIV antibody detection using ADVIA Centaur® HIV 1/O/2 Enhanced (Siemens) following the manufacturer’s instructions.

### Statistical analysis

All analyses were performed using IBM SPSS Statistics for Windows, Version 21.0 (IBM Corp, Armonk, NY, USA) and OpenEpi^35^.

The results obtained through DBS were compared with those obtained through venipuncture at HUIL (Gold Standard). The sensitivity, specificity, positive predictive values (PPV), negative predictive values (NPV), positive likelihood ratios (PLR), and negative likelihood ratios (NLR) were calculated for each of the techniques.
